# A126 IMPACT OF TIME OF DAY ON PROCEDURAL OUTCOMES IN ENDOSCOPIC RETROGRADE CHOLANGIOPANCREATOGRAPHY (ERCP): ANALYSIS FROM A TERTIARY REFERRAL CENTER

**DOI:** 10.1093/jcag/gwad061.126

**Published:** 2024-02-14

**Authors:** M Deeb, N Sabrie, K Khalaf, N Calo, J Mosko, N Forbes, S Grover

**Affiliations:** University of Toronto, Toronto, ON, Canada; University of Toronto, Toronto, ON, Canada; Division of Gastroenterology, St Michael's Hospital, Toronto, ON, Canada; Division of Gastroenterology, St Michael's Hospital, Toronto, ON, Canada; Division of Gastroenterology, St Michael's Hospital, Toronto, ON, Canada; University of Calgary, Calgary, AB, Canada; Division of Gastroenterology, St Michael's Hospital, Toronto, ON, Canada

## Abstract

**Background:**

Endoscopic retrograde cholangiopancreatography (ERCP) is an established diagnostic and therapeutic tool for hepatobiliary disease. Given its technical demands, it remains one of the highest-risk endoscopic procedures. Addressing modifiable factors, such as operator fatigue, may mitigate procedural risk. In colonoscopy, there is conflicting data on whether procedure time of day, as a surrogate of operator fatigue, affects outcomes, with some literature demonstrating decreased procedure completion and polyp detection rates in the afternoon. There is a paucity of data evaluating this potential relationship in ERCP.

**Aims:**

To evaluate the impact of procedure time of day on procedural success and short-term adverse outcomes in patients undergoing ERCP.

**Methods:**

A retrospective review of ERCP's performed on adult patients at our tertiary referral center from January 1, 2011 to December 31, 2020 was performed. The primary outcome was the procedural success rate, defined as successful navigation to the papilla, selective duct cannulation and cholangiography, and realization of the intended therapeutic goals. Secondary outcomes included procedure duration, rate of deep ductal cannulation, rate of sphincterotomy, and short-term (30-day) adverse events (immediate bleeding, delayed bleeding, pancreatitis, perforation). Statistical analysis was conducted using R. Categorical variables were compared using the Chi-square test of independence or Fisher’s exact test. Continuous variables were compared using T-tests or the Mann-Whitney-U test.

**Results:**

A total of 5755 ERCP’s were performed between 8 AM – 6 PM; 2863 were performed before 12PM (AM group) and 2892 after 12PM (PM group). Baseline characteristics were similar between the two cohorts, with the exception of hypertension (33.7% AM vs 30.1% PM; p=0.003), and anticoagulation (20.3% AM vs 18.3% PM; p=0.05). In both groups, the most common ERCP indication was choledocholithiasis. The primary operators in both cohorts were clinical fellows. There was no difference in procedural success rate (87.0 % AM vs 86.7% PM; p = 0.72), procedure duration (33.6 minutes AM vs 32.9 minutes PM; p = 0.29), rates of deep cannulation (82.6% AM vs 83.0% PM; p = 0.81) and sphincterotomies (63.2% vs 62.7%; p = 0.68). Rate of adverse events were similar, with slightly higher rates of immediate bleeding in the AM group (5.0% AM vs 3.8% PM; p = 0.03). Results were similar in a subgroup analysis of patients with altered anatomy.

**Conclusions:**

In this large retrospective review of ERCPs performed at a tertiary referral center, the procedure time of day did not impact procedural success rate. There were slightly higher rates of immediate bleeding in the AM group, though this may be explained by higher rates of anticoagulation in that group.

**Funding Agencies:**

None

